# The use of self-report questions to examine the prevalence of musculoskeletal problems: a test-retest study

**DOI:** 10.1186/s12891-016-0946-6

**Published:** 2016-02-24

**Authors:** Tiffany K. Gill, Graeme R. Tucker, Jodie C. Avery, E. Michael Shanahan, Hylton B. Menz, Anne W. Taylor, Robert J. Adams, Catherine L. Hill

**Affiliations:** NHMRC Early Career Fellow, School of Medicine, Faculty of Health Sciences, The University of Adelaide, Level 7, SAHMRI, North Tce, Adelaide, SA 5000 Australia; School of Medicine, Faculty of Health Sciences, The University of Adelaide, Adelaide, SA 5005 Australia; Population Research and Outcome Studies, Discipline of Medicine, Faculty of Health Sciences, The University of Adelaide, Adelaide, SA 5005 Australia; Rheumatology Department, Southern Adelaide Health Service, Repatriation General Hospital, Daws Rd, Daw Park, SA 5042 Australia; School of Medicine, Flinders University, Bedford Park, SA 5041 Australia; School of Allied Health, College of Science, Health and Engineering, La Trobe University, Bundoora, Vic 3083 Australia; The Health Observatory, The Queen Elizabeth Hospital, The University of Adelaide, Adelaide, SA 5005 Australia; Rheumatology Department, The Queen Elizabeth Hospital, Woodville Rd, Woodville, SA 5011 Australia

**Keywords:** Musculoskeletal pain, Test retest, Reliability, Joint pain, Prevalence

## Abstract

**Background:**

Case definition has long been an issue for comparability of results obtained for musculoskeletal pain prevalence, however the test-retest reliability of questions used to determine joint pain prevalence has not been examined. The objective of this study was to determine question reliability and the impact of question wording, ordering and the time between questions on responses.

**Methods:**

A Computer Assisted Telephone Interviewing (CATI) survey was used to re-administer questions collected as part of a population-based longitudinal cohort study. On two different occasions questions were asked of the same sample of 203 community dwelling respondents (which were initially randomly selected) aged 18 years and over at two time points 14 to 27 days apart (average 15 days). Reliability of the questions was assessed using Cohen’s kappa (κ) and intraclass correlation coefficient (ICC) and whether question wording and period effects existed was assessed using a crossover design.

**Results:**

The self-reported prevalence of doctor diagnosed arthritis demonstrated excellent reliability (κ = 0.84 and κ = 0.79 for questionnaires 1 and 2 respectively). The reliability of questions relating to musculoskeletal pain and/or stiffness ranged from moderate to excellent for both types of questions, that is, those related to ever having joint pain on most days for at least a month (κ = 0.52 to κ = 0.95) and having pain and/or stiffness on most days for the last month (κ = 0.52 to κ = 0.90). However there was an effect of question wording on the results obtained for hand, foot and back pain and/or stiffness indicating that the area of pain may influence prevalence estimates.

**Conclusions:**

Joint pain and stiffness questions are reliable and can be used to determine prevalence. However, question wording and pain area may impact on estimates with issues such as pain perception and effect on activities playing a possible role in the recall of musculoskeletal pain.

## Background

Recent work, undertaken as part of the Global Burden of Disease Study 2010, has highlighted the impact of musculoskeletal conditions worldwide [1–7]. However, a major issue identified as a result of this work was that of case definition. Variation exists across studies in terms of the prevalence period and the lack of standardised and valid questions asked as part of population-based surveys, which impacts on the ability to capture total disease burden [8]. This has been a longstanding issue, also highlighted by previous authors such as Picavet and Hazes [9] and Bombard et al. [10].

Other issues which are likely to impact on prevalence estimates obtained from questionnaires include response category wording and mode of administration. Questionnaire wording has been shown to influence the reported prevalence of conditions such as wheezing in asthma [11], and in terms of musculoskeletal pain, the provision of different anatomical descriptions of the back provided different prevalence estimates for low back pain [12]. However the wording of questions related to illness burden was not shown to impact responses [13]. With respect to mode of data collection, Feveile et al. [14] determined using a randomized trial that the mode of data collection (mailed questionnaire versus telephone) impacted on the response patterns for self-assessed health items, and the use of online and face-to-face surveys affect responses to burden of illness questions [13]. This view is also supported by Bowling [15] who suggested that data quality may be influenced by the questionnaire administration mode and this difference may affect the answers provided to the questions. However, although differences according to mode of administration may exist, these may be dependent on the type of mode and the conditions that are assessed. An example is that web and paper-based modes showed no differences when assessing health related quality of life among men with prostate cancer [16] and manikins and written questions showing similar results for musculoskeletal conditions [17].

It is essential that data used in the planning and monitoring of health services and disease prevalence in populations, are as accurate as possible [18], particularly when collected on different occasions. Using common measures in population studies has the advantage of comparability of data across populations [19]. The accuracy or precision of survey questions can be measured and bias minimised by assessing their reliability [20].

One way of assessing reliability is by using a test-retest methodology where responses to questions are assessed in the same group of people, after a specified time period, to see if they provide similar results. This is especially important for questions that are used in regular or ongoing surveys. The reliability of questions in telephone health survey questionnaires, such as the Behavioral Risk Factor Surveillance System (BRFSS) in the United States, has been addressed in the literature [18, 21–25]. A range of demographic variables and health risk factors from the BRFSS questionnaires were investigated using reliability tests. Variables with the highest reproducibility included demographic variables as well as self-reported health. Health risk factors and ‘poor’ health days were found to be slightly less reliable, although still at an acceptable level [18, 21–24]. A study of the South Australian Monitoring and Surveillance System (SAMSS) demonstrated that in this population, the presence of the majority of self-reported chronic conditions demonstrated substantial to almost perfect agreement while demographic questions showed high reliability, and the reliability of questions relating to self-reported risk factors ranged from excellent to moderate agreement [26].

In population studies, test-retest methods have been used to assess the reliability of different questionnaire tools associated with musculoskeletal pain. Examples include: Balogh et al. [27] who examined occupational exposure of the shoulder and neck region in relation to shoulder and neck pain development and repeated the questions at 12 months; Dziedzic et al. [19] who assessed the test-retest reliability of the Australian/Canadian Osteoarthritis Hand Index at a one month interval; Haldorsen et al. [28] who undertook an assessment of the Disabilities of the Arm, Shoulder and Hand (DASH) questionnaire approximately one week apart among those with shoulder impingement; and Harris et al. [29] who assessed the reliability of responses to the Oxford Knee Score obtained a few days apart, among patients undergoing conservative treatment for knee osteoarthritis. There are generally fewer studies that have assessed the reliability of prevalence questions. Bombard et al. [10] demonstrated that the self-reported prevalence of arthritis obtained from the BRFSS was high using the question “Have you ever been told by a doctor that you have arthritis?” and Dal Grande et al. [26] demonstrated excellent reliability using the identical question.

Picavet and Hazes [9] also examined the prevalence of specific self-reported doctor diagnosed musculoskeletal diseases in a general population (back herniated disc, gout, repetitive strain injury, epicondylitis, osteoarthritis of knee and hip, osteoporosis, whiplash, rheumatoid arthritis, other chronic arthritis, fibromyalgia and tendinitis/capsulitis) at baseline and six months, and demonstrated good reliability for all conditions except repetitive strain injury (non-specific arm pain) and chronic arthritis, which were fair to moderate. However, while the prevalence of non-specific areas of pain have been reported by various authors (shoulder, elbow, wrist and hand pain [30]; upper limb pain [9]; distal arm pain [31]; hip [32]; hip and knee pain [33, 34]; hip, knee and foot pain [35]; foot and ankle [36]; neck pain and back pain [37]; back pain [36]), there are few studies which examine the reliability of these questions used to assess musculoskeletal pain prevalence.

The aims of this study were to examine (i) the test-retest reliability of self-reported doctor diagnosed arthritis and non-specific musculoskeletal pain in six areas of the body and (ii) the effect of questionnaire wording, order and time between questionnaires.

## Methods

The North West Adelaide Health Study (NWAHS) is a representative longitudinal study of 4056 randomly selected adults aged 18 years and over at the time of recruitment from the north-west region of Adelaide, South Australia. The sample region represents approximately half of the metropolitan area (total population of approximately 1.3 million) and almost one-third of the population in South Australia (population of approximately 1.7 million), which has the second highest elderly population of all the Australian states and territories [38]. The aim of the study is to provide longitudinal measured and self-reported data to assist in increasing the ability of strategies and policies to prevent, detect and manage a range of chronic conditions [39]. The study commenced in 1999–2003 (Stage 1), Stage 2 was conducted between 2004 and 2006 and Stage 3 was conducted between 2008 and 2010.

### Questions related to the prevalence of musculoskeletal conditions and arthritis

A Computer Assisted Telephone Interview (CATI), a self-completed questionnaire and a clinic assessment has been used at each stage [39, 40] in order to collect data. In Stage 2, the prevalence of musculoskeletal conditions was determined using the CATI. In Stage 3, due to questionnaire lengths, the prevalence of musculoskeletal conditions was determined using the CATI (for shoulder pain and arthritis) and the self-complete questionnaire (for back, hip, knee, foot, hand pain). The self-complete questionnaire was mailed to participants, however where possible, they were asked to return the questionnaire at the clinic visit to enable checking of responses by clinic staff. The questions that were used are summarised in Table 1, with the questions in Stage 3, using the wording “pain in the last month” aimed at determining the presence of current pain.

**Table 1:** Summary of questions

Questionnaire 1	Response options	Questionnaire 2	Response options
Have you ever had pain or aching in your low back, either at rest or when moving, on most days for at least a month?	Yes	Over the past month, have you had pain or aching in your low back, either at rest or when moving, on most days?	Yes
	No		No
	Don’t know/ refused		Don’t know/ refused
Have you ever had stiffness in your low back, when first getting out of bed in the morning, on most days for at least a month?	Yes	Over the past month, have you had stiffness in your low back, when first getting out of bed in the morning, on most days?	Yes
	No		No
	Don’t know/ refused		Don’t know/ refused
Have you ever had pain or aching in your hips, either at rest or when moving, on most days for at least a month?	Yes	Over the past month, have you had pain or aching in your hips, either at rest or when moving, on most days?	Yes, Left hip
	No		Yes, Right hip
	Don’t know/ refused		No
			Don’t know/ refused
Have you ever had stiffness in your hip joints or muscles, when first getting out of bed in the morning, on most days for at least a month?	Yes	Over the past month, have you had stiffness in your hip joints or muscles, when first getting out of bed in the morning, on most days?	Yes, Left hip
	No		Yes, Right hip
	Don’t know/ refused		No
			Don’t know/ refused
Have you ever had pain, aching or stiffness in your knees, either at rest or when moving, on most days for at least a month?	Yes	Over the past month, have you had pain, aching or stiffness in your knees, either at rest or when moving, on most days?	Yes, Left knee
	No		Yes, Right knee
	Don’t know/ refused		No
			Don’t know/ refused
On most days, do you have pain, aching or stiffness in either of your feet?	No	Over the past month, have you had pain, aching or stiffness in either of your feet on most days?	No
	Yes, left foot		Yes, Left foot
	Yes, right foot		Yes, Right foot
	Yes, both feet		Not applicable (eg amputee)
	Yes, not sure what side		Don’t Know
	Not applicable (eg amputee)		
	Don’t know		
Have you ever had pain or aching in your shoulder, either at rest or when moving, on most days for at least a month?	Yes	Over the past month, have you had pain or aching in either or both of your shoulders, either at rest or when moving, on most days?	Yes
	No		No
	Don’t know/ refused		Don’t know/ refused
Have you ever had stiffness in your shoulder, when first getting out of bed in the morning, on most days for at least a month?	Yes	Over the past month, have you had stiffness in either or both of your shoulders, when first getting out of bed in the morning, on most days?	Yes
	No		No
	Don’t know/ refused		Don’t know/ refused
Have you had pain, aching or stiffness in your hands, either at rest or when using them, on most days for at least a month?	Yes	Over the past month, have you had pain or aching in your hands, either at rest or when moving, on most days?	Yes, Left hand
	No		Yes, Right hand
	Don’t know/ refused		No
			Don’t know / refused
		Over the past month, have you had stiffness in your hands when first getting out of bed in the morning, on most days?	Yes, Left hand
			Yes, Right hand
			No
			Don’t know / refused
Have you ever been told by a doctor that you have arthritis?	Osteoarthritis	Have you ever been told by a doctor that you have arthritis?	Osteoarthritis
	Rheumatoid arthritis		Rheumatoid arthritis
	Yes, other (specify)		Yes, other (specify)
	Yes, don’t know type		Yes, don’t know type
	No, don’t have arthritis		No, don’t have arthritis
	Don't know / refused		Don't know / refused

As a result of the different time frames used for prevalence across the stages (most days for at least a month vs. over the past month…on most days), it was decided to conduct a study in order to:

i. Determine the test-retest reliability of each of the questions at two time points
ii. Determine whether the questions asked in the different stages of the NWAHS could be combined in order to provide longitudinal prevalence estimates and comparison of changes in prevalence over time.

**Fig. 1:**
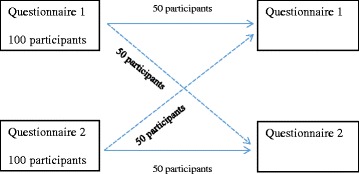
Allocation of participants to each questionnaire

In order to achieve this, a separate random sample of community dwelling adults aged 18 years and over was contacted by telephone by a health survey research company. Potential participants were broadly informed of the nature of the research (that is, it was a survey on musculoskeletal pain) and that they would be contacted on two different occasions (at the current time and in approximately two weeks’ time) in order to test questions so as to provide the best information about joint pain. If participants gave their consent, the short survey was commenced. Participants were randomly assigned to one of four groups: (i) those who were asked the musculoskeletal questions from the NWAHS Stage 2 (questionnaire 1) twice, (ii) those who were asked the questions from Stage 3 (questionnaire 2) twice, (iii) those who were asked questionnaire 1 and then questionnaire 2 at the second interview and (iv) those who were asked questionnaire 2 and then questionnaire 1. A diagram detailing the order of the questionnaires is presented in Fig. 1.

Overall, 52 participants provided responses at time 1 and 2 for questionnaire 1, 51 participants provided responses at the two time points for questionnaire 2 and 50 participants each provided responses at time 1 and 2 for questionnaire 1 then 2 and for questionnaire 2 then 1. Thus responses were obtained from 203 respondents in total.

### Statistical analyses

Statistical analyses were conducted using G*Power [41], to determine power and STATA version 13.1 (StataCorp, College Station, TX, USA). As the question relating to self-reported arthritis was asked of all participants on both occasions, the prevalence reported is that obtained on the first contact (*n* = 203). In order to obtain the prevalence of musculoskeletal pain as determined by the questions used at Stage 2 of the NWAHS, the response obtained from all participants that answered questionnaire 1 first were combined with the response from participants who answered questionnaire 1 after questionnaire 2 (*n* =152). To determine the prevalence of musculoskeletal pain using the questions from Stage 3 of the NWAHS, the response obtained from all participants who answered questionnaire 2 first were combined with the response from participants who answered questionnaire 2 after questionnaire 1 (*n* =151). The prevalence obtained from questionnaire 1 and 2 individually (*n* =102 and *n* =101 respectively) was also determined.

Cohen’s kappa (κ) [42, 43] was used to assess the agreement in responses for those who were asked either questionnaire 1 (*n* =52) or questionnaire 2 (*n* =51) at the first and second telephone call and also to assess the agreement between time 1 and time 2 for the arthritis prevalence questions, which was asked of all respondents (*n* =203). Reliability values of between 0.81 and 1.00 were considered to be “excellent” agreement, between 0.61 and 0.80 “good” agreement, between 0.41 and 0.60 “moderate”, between 0.21 and 0.40 “fair” and less than 0.20 were considered “poor” agreement [44]. For each variable, the percentage of observed agreement and expected agreement (the level of agreement expected by chance) was also calculated.

Secondly the pkcross command in STATA [45] was used, as 100 participants were asked one form of the musculoskeletal prevalence questions and then asked the other form on the second occasion, in a crossover study design. Because of the differences in wording between the two questionnaires, the pkcross command was used to determine whether the “treatment” (wording of prevalence questions), the order in which questions were asked and/or the period (timeframe between questions) impacts on the responses provided, using an analysis of variance for a crossover study. The responses used in the analysis were those who answered questionnaire 1 at time 1 and questionnaire 2 at time 2 and those who responded to questionnaire 2 at time 1 and questionnaire 1 at time 2. As the hip, knee, foot and hand pain questions in questionnaire 2 specified right and left sides, these responses were combined into a single yes/no variable for comparison purposes.

### Ethical approval

Ethical approval for the study was obtained from the Human Research Ethics Committee of the University of Adelaide (H-2012-098). All participants provided informed consent.

## Results

Overall a complete response (that is, questionnaires completed on two occasions) was obtained from 203 respondents. There was an average of 15 (SD 1.7) days between each questionnaire (range 14–27 days). The mean age of participants was 60 years (range 19–91) and 60.6 % were female. There was complete agreement between the two time points in responses to sex and age (κ = 1.00 and ICC = 1.00 respectively). While the final sample size was limited by time and costs, the retrospective power calculation indicated that with a sample size of 100, the achieved power for a McNemar test to detect an odds ratio of 2 ranged between 20.9 and 62.4 %.

The results of the reliability testing for the arthritis question are presented in Table 2 and in Table 3, for questionnaire 1. Kappa values ranged between moderate to excellent agreement, with the lowest level of agreement for hand pain, aching or stiffness, shoulder pain or aching and shoulder stiffness on most days for at least a month. For questionnaire 2, kappa values ranged between fair to excellent agreement with the lowest values for hand stiffness on most days over the last month (Table 4).

**Table 2:** Arthritis prevalence, kappa and percent agreement for those asked Questionnaire 1 and 2 at two different times

	Overall prevalence (%)	Prevalence Ques 1 (%)	Prevalence Ques 2 (%)	Ques 1 time 1 and 2 (*N* =52)			Ques 2 time 1 and 2 (*N* =51)		
Arthritis	*N* =203	*N* =102	*N* =101	% agreement	% expected agreement	kappa	% agreement	% expected agreement	kappa
Osteoarthritis	21.7	22.5	20.8	92.3	73.9	0.71	94.1	67.3	0.82
Rheumatoid arthritis	1.5	1.0	2.0	100.0	-	1.00	100.0	-	1.0
Other type arthritis	2.5	3.9	1.0	98.1	94.4	0.66	100.0	-	1.0
Don’t know type	14.3	14.7	13.9	86.5	65.5	0.61	88.2	76.0	0.51
No arthritis	61.1	57.8	64.4	88.5	51.2	0.76	90.2	53.3	0.79
Arthritis overall	38.9	42.2	35.6	92.0	51.2	0.84	90.2	53.3	0.79

**Table 3:** Prevalence, kappa and percent agreement for those asked Questionnaire 1 at two different times

	Overall prevalence (%)	Prevalence Ques 1 time 1 (%)	Ques 1 time 1 and 2 (*N* =52)		
	*N* =152	*N* =102	% agreement	% expected agreement	kappa
Ever had pain or aching in your low back, on most days for at least a month^a^	44.1	47.1	86.3	53.8	0.70
Ever had stiffness in your low back, on most days for at least a month^a^	34.2	34.3	98.0	59.3	0.95
Ever had pain or aching in your hips, on most days for at least a month	27.0	27.5	86.5	61.1	0.65
Ever had stiffness in your hip joints or muscles, on most days for at least a month^a^	25.0	25.0	86.3	61.1	0.65
Ever had pain, aching or stiffness in your knees, on most days for at least a month^a^	32.2	35.3	86.3	60.9	0.65
On most days, do you have pain, aching or stiffness in either of your feet	28.3	29.4	90.4	59.9	0.76
Hand pain, aching or stiffness either at rest or when moving on most days for at least a month	36.8	40.2	78.9	54.0	0.54
Ever had pain or aching in your shoulder, on most days for at least a month	37.5	37.3	76.6	51.9	0.52
Ever had stiffness in your shoulder, on most days for at least a month^a^	19.7	18.6	86.5	67.3	0.59

^a^Don’t know responses removed from the kappa analysis

**Table 4:** Prevalence, kappa and percent agreement for those asked Questionnaire 2 at two different times

	Overall prevalence (%)	Prevalence Ques 2 time 1 (%)	Ques 2 time 1 and 2 (*N* =51)		
	*N* =151	*N* =101	% agreement	% expected agreement	kappa
Low back pain on most days over the past month	39.7	42.6	76.5	51.1	0.52
Low back stiffness on most days over the past month	26.5	30.7	92.2	61.7	0.80
Hip pain on most days in the last month (left)	15.2	17.8	88.2	73.5	0.56
Hip pain on most days in the last month (right)	13.3	15.8	90.2	67.3	0.70
Hip pain on most days in the last month (no)	78.2	75.3	82.4	61.1	0.55
Hip stiffness on most days in the last month (left)	12.6	11.9	94.1	77.7	0.74
Hip stiffness on most days in the last month (right)	11.9	12.9	92.2	73.5	0.70
Hip pain on most days in the last month (no)	82.8	83.2	92.2	68.4	0.75
Knee pain, aching or stiffness either at rest or when moving on most days over the last month (left)	20.5	19.8	92.2	70.6	0.73
Knee pain, aching or stiffness either at rest or when moving on most days over the last month (right)	23.2	24.8	90.2	65.1	0.72
Knee pain, aching or stiffness either at rest or when moving on most days over the last month (no)	70.2	71.3	88.2	60.1	0.71
Foot pain, aching or stiffness in either feet on most days over the last month (left)	17.9	16.8	92.2	76.2	0.67
Foot pain, aching or stiffness in either feet on most days over the last month (right)	17.9	19.8	88.2	73.2	0.56
Foot pain, aching or stiffness in either feet on most days over the last month (no)	78.2	79.2	88.2	73.2	0.56
Pain or aching in your hands on most days over the last month (left)	23.8	24.8	90.2	64.9	0.72
Pain or aching in your hands on most days over the last month (right)	28.5	31.7	90.2	56.1	0.78
Pain or aching in your hands on most days over the last month (no)	70.2	67.3	90.2	56.1	0.78
Stiffness in your hands on most days over the last month (left)	12.6	12.9	92.2	85.5	0.46
Stiffness in your hands on most days over the last month (right)	13.9	14.9	88.2	79.2	0.43
Stiffness in your hands on most days over the last month (no)	84.5	83.2	86.3	77.7	0.38
Pain or aching in either or both shoulders on most days over the past month^a^	29.8	31.7	96.1	60.2	0.90
Stiffness in either or both shoulders on most days over the past month	17.2	19.8	90.2	69.7	0.68

^a^Don’t know responses removed from the kappa analysis

The results of the crossover study are shown in Table 5 and indicate that the back pain and stiffness questions and the hand pain/stiffness questions were impacted by question wording and the time between questionnaires and the foot pain/stiffness questions were impacted by question wording. There was no impact of the sequence of questionnaires on responses.

**Table 5:** Effect of sequence, questionnaire wording and time between questionnaires on prevalence questions in questionnaire 1 and questionnaire 2

	Sequence effect	Question wording effect	Time between questionnaires
	*p*-value	*p*-value	*p*-value
Back pain^a^	0.326	0.016	0.038
Back stiffness^a^	0.620	0.009	0.028
Hip pain	0.593	0.111	0.593
Hip stiffness^a^	0.889	0.072	0.795
Knee pain/stiffness^a^	0.233	0.346	0.159
Foot pain/stiffness	0.885	0.039	0.443
Hand pain/stiffness^a^	0.631	0.026	0.002
Shoulder pain	0.363	0.292	0.292
Shoulder stiffness	0.566	0.111	0.291

^a^Don’t know responses removed from the analysis

It must be noted that the prevalence results provided in Table 2 for arthritis, Table 3 for the questions in questionnaire 1 and in Table 4 for the questionnaire 2 questions were not weighted to the population as all other prevalence estimates obtained from the NWAHS have been previously, and need to be interpreted with caution.

## Discussion

Issues of case definition and variation across studies that examine the prevalence of musculoskeletal disorders have long been an issue for researchers and policy makers alike [9]. The lack of standardized questions makes it difficult to compare studies and also impacts on the ability to truly highlight the scale of musculoskeletal conditions within the population worldwide. This study aimed to use the different prevalence questions asked at two different time points of a longitudinal, population base cohort study, in a random community sample in order to examine the reliability of questions and the potential impact of question wording and sequencing on responses. The results indicated that prevalence questions are reliable, however question wording and the location of the pain may influence prevalence estimates and interpretation of results.

In line with previous reliability studies conducted in South Australia [26], age and sex demonstrated a high degree of reliability (ICC = 1.0 and κ = 1.0, respectively). This would be expected as protocols were put in place to ensure that the same respondent was interviewed on each occasion, thus providing excellent agreement in the responses for these two characteristics.

The overall prevalence of arthritis variable has been shown to have good reliability in previous work [26]. It is also of note that the individual types of arthritis demonstrated good to excellent agreement although “don’t know” the type of arthritis had the lowest levels of agreement. The most common form of arthritis is osteoarthritis and there may have been some respondents who varied particularly between the “don’t know type of arthritis” and the “osteoarthritis” categories at the different survey times. However, this generally indicates that participants do recall being told that they have a health condition and the questions that are used to obtain a prevalence estimate for arthritis are reliable. This has also been shown by Bombard et al. [10] who determined a high reliability for doctor diagnosed arthritis (κ = 0.76).

The questions relating to “ever” having back pain or stiffness on most days for at least a month demonstrated good and excellent agreement beyond chance respectively. However questions relating to back pain on most days over the past month had only moderate reliability. While respondents were likely to remember if they had “ever” had back pain or stiffness, the presence of pain in the last month in particular could however have been impacted by the time between each of the surveys and this may be reflected in the significant period effect also obtained from the crossover analysis. Back stiffness also demonstrated a significant period effect and there was a significant effect as a result of the change in question wording for both back pain and stiffness. While the timeframe between questionnaires was only approximately two weeks, the period effect obtained for the back pain and stiffness questions appears to indicate a recall bias may exist.

Ever having hip pain or stiffness demonstrated good agreement as did having hip pain or stiffness in the last month although there was a difference between the right and left sides in the level of agreement. Ever having knee pain, aching or stiffness and knee pain, aching or stiffness in the last month had good reliability, ever having shoulder pain or stiffness demonstrated moderate reliability and shoulder pain in the last month demonstrated excellent reliability and stiffness good reliability. It may be that some for some joint areas it is easier to recall current pain and the relative impact on activities of daily life may also influence the recollection of joint pain. However, the crossover analysis indicated no impact of question wording on these results.

The foot pain questions were “On most days, do you have pain, aching or stiffness in either of your feet?” and “Over the past month, have you had pain, aching or stiffness in either of your feet on most days?” The reliability for the first question was good, however the reliability for the second question ranged between good and moderate, possibly because it requires participants to place their symptoms within a time frame of one month. The first question also does not provide a specific time frame over which the foot pain, aching or stiffness is present and this was reflected in a significant effect of questionnaire wording on the estimates as a result of the crossover analysis.

Finally having hand pain, aching in the last month demonstrated good reliability, while stiffness demonstrated fair to moderate reliability. The first question combined pain, aching or stiffness and also, like the foot question, did not include the word “ever”. The questions relating to prevalence in the past month separated pain and aching and stiffness and these variables had to be combined for analysis purposes. Consequently there was a significant effect of question wording on responses for hand pain and stiffness and also an effect of the time between questions.

The prevalence estimates of musculoskeletal conditions obtained in this community sample were generally high. The introduction to the survey did indicate that the questions would be about pain in the joints and thus those with joint pain may have been more likely to continue the survey. While the prevalence of back pain was within the range of one month prevalences described by Hoy et al. [46] of 24.0 % to 49.5 %, the prevalence of foot, shoulder and hand pain were all higher than that previously described from the North West Adelaide Health Study [47–50]. The proportion of those with self-reported doctor diagnosed arthritis was also higher than that previously reported for the South Australian population by Dal Grande et al. [26]. However the aim of this study was to compare reliability of responses and question design rather than to obtain prevalence estimates per se. Any prevalence estimates obtained from this study were not weighted to the population and thus have limited generalizability and need to be interpreted with caution.

A possible limitation of the study was the lack of facial and/or body cues, as a result of using the telephone, which may indicate misunderstanding or incorrect interpretation of a question. In order to address this; highly skilled telephone interviewers who undertake many health-related telephone surveys were used. Consequently, they have the experience to detect potential question misunderstandings and provide clarification if needed. This study also used questions from the NWAHS, which are always pilot tested prior to the conduct of the main study.

Other limitations of this study are the small number of respondents (*n* =203) and while the study was broadly representative in terms of the age of participants, there was a slightly higher proportion of females interviewed compared to the NWAHS and also a higher prevalence of those reporting musculoskeletal problems. Thus there is the potential for selection bias of participants; those with musculoskeletal problems may have been more likely to participate. However, again, because the focus of the study was the reliability of questions this is likely to have little impact. The timeframe of an average of 15 days (SD 1.7) was chosen as participants were unlikely to remember their first responses but it was also unlikely that answers would change significantly. However, there are changes that could have occurred between interviews particularly with regard to the timeframe of the presence of pain on most days within the last month, which may impact of the reliability of responses. Recall bias in terms of “ever” having experienced joint pain may also have been an issue. Survey responses have also been shown to be effected by mode of collection [13–15]. This study only used the telephone as the mode of data collection, however the use of a different mode (for example self-complete questionnaire) may impact on the reliability of questions and also the results of the crossover study. In addition, as stated above, the prevalence estimates need to be interpreted with caution.

The results indicate that generally the reliability of the questions used to assess the prevalence of musculoskeletal disorders ranges from moderate to excellent and are therefore suitable to use to determine arthritis and joint pain prevalence. This is in spite of the fact that pain (of any kind) “in the last month” can change over time quite legitimately, whereas changes in “ever having pain” are not legitimate if the response changes from “yes” to “no”. Consequently, it can be argued that the issue of reliability essentially only applies to questionnaire 1. The issue that remains is that of case definition or which prevalence estimate is most appropriate to use. The generally high reliability of questions relating to pain over the last month perhaps could be considered more of an indicator that these conditions tend to last longer than a month. The absence of question wording effect means that the nature of the complaint does not differ between “ever” and “last month”. Thus, either question wording should elicit the same response, so the questions can be considered equivalent subject to the caveat above. It also appears that there may not be a significant effect of question wording on the prevalence estimates for some joint areas (shoulder, hip, knee).

However for other areas of pain (foot, hand, back) question wording has a significant impact and for two of these areas (hand and back) there was also a significant effect of the time period between the responses. While questionnaire wording has been shown to have an effect on survey responses [12], these appear to depend on the type of questions that are asked [11, 13]. It is unclear however as to why an effect exists for the back, foot and hand as opposed to the shoulder, knee and hip and also why an effect of time or possibly recall bias is present for the hand and back in particular. Further work is required to elucidate the reasons behind this but it may in part be due to the impact of that particular joint pain or stiffness on function and activities of daily living, the nature of the joint pain itself (as the level of pain or stiffness was not assessed), chronicity and participant’s perception of their pain.

## Conclusion

Joint pain and stiffness questions are reliable and can be used to determine prevalence. However, question wording and pain location may influence prevalence estimates and impacts on the interpretation, the relevance of case definition and any analyses that may be undertaken.

## Availability of supporting data

Data from this study form part of the NWAHS. As this is an ongoing cohort study, these data are available on application to the chief investigators. Details are available at: http://health.adelaide.edu.au/pros/data/nwahs/

## Abbreviations

BRFSS: Behavioral Risk Factor Surveillance System
CATI: computer assisted telephone interviewing
CI: confidence interval
DASH: Disabilities of the Arm, Shoulder and Hand
ICC: intraclass correlation coefficient
NWAHS: North West Adelaide Health Study
SAMSS: South Australian Monitoring and Surveillance System
SD: standard deviation
Κ: Cohen’s kappa
